# Combination of Dll4/Notch and Ephrin-B2/EphB4 targeted therapy is highly effective in disrupting tumor angiogenesis

**DOI:** 10.1186/1471-2407-10-641

**Published:** 2010-11-23

**Authors:** Dusan Djokovic, Alexandre Trindade, Joana Gigante, Marina Badenes, Lilliana Silva, Ren Liu, Xiuqing Li, Ming Gong, Valery Krasnoperov, Parkash S Gill, Antonio Duarte

**Affiliations:** 1 Centro Interdisciplinar de Investigação em Sanidade Animal (CIISA), Lisbon Technical University, Lisbon, Portugal; 2 Instituto Gulbenkian de Ciência, Oeiras, Portugal; 3 Department of Pathology, University of Southern California, Los Angeles, USA; 4 Department of Medicine, University of Southern California, Los Angeles, USA; 5 Vasgene Therapeutics, Los Angeles, CA, USA

## Abstract

**Background:**

Dll4/Notch and Ephrin-B2/EphB4 pathways play critical roles in tumor vessel development and maturation. This study evaluates the efficacy of the inhibition of both signaling pathways, alone and in combination, in reducing the growth of an autochthonous mouse tumor and assesses potential adverse effects.

**Methods:**

We used the transgenic RIP1-Tag2 tumor model to study the effects of 1) inhibition of Dll4/Notch by either *Dll4 *allelic deletion or use of a soluble extracellular Dll4 (sDll4), 2) inhibition of Ephrin-B2/EphB4 signaling by a soluble extracellular EphB4 fused to albumin (sEphB4-Alb), and 3) inhibition of both pathways by sEphB4-Alb combined with either *Dll4 *allelic deletion or sDll4. To investigate adverse effects, we used inducible endothelial-specific *Dll4 *knock-out mice, treated with sEphB4-Alb, and carried out histopathological analysis.

**Results:**

*Dll4 *allele deletion or soluble Dll4 treatment resulted in increased tumor vessel density, reduced mural cell recruitment and vessel perfusion which resulted in reduced tumor size. The soluble EphB4 instead reduced vessel density and vessel perfusion, leading to reduction of tumor size. Greater efficacy was observed when sEphB4-Alb was combined with either *Dll4 *allele deletion or sDll4 in regards to tumor size, vessel perfusion and mural cell recruitment. Induced endothelial specific *Dll4 *loss-of-function caused hepatic vascular alterations, which were prevented by concomitant sEphB4-Alb treatment.

**Conclusion:**

Combination targeting of Dll4/Notch and Ephrin-B2/EphB4 has potential for clinical investigation, providing cumulative efficacy and increased safety over Dll4/Notch inhibition alone.

## Background

Targeting tumor angiogenesis, in particular through blocking vascular endothelial growth factor (VEGF) activity, has been successful, with several drugs now approved for use in many different cancer types [1]. Rapid development of resistance to these [2], however, highlights the need for other vascular targeted therapies.

Dll4/Notch pathway, which plays prominent role in angiogenesis, has become such a target. Dll4 is critical for embryonic vascular development and arterial specification and is markedly induced in murine and human tumor vessels [3-8]. Notch1 and Notch4 are also expressed in tumor vessels [6]. Notch ligand expression and Notch activation is induced by VEGF [9,10]. Dll4/Notch signaling in turn attenuates VEGF signaling, thus arresting endothelial cell proliferation, followed by recruitment of mural cells and vessel maturation [7-9,11,12].

Not surprisingly, targeted *Dll4 *allele deletion results in increased vascular proliferation but, unexpectedly, impaired vessel structure and function [8]. Overall, inhibition of Dll4/Notch causes reduced tumor growth. Careful evaluation of the tumor vessels reveals increased vessel proliferation, reduced lumen size, reduced mural cell recruitment, increased leakiness and reduced perfusion. Furthermore, tumors resistant to VEGF targeted therapy remain responsive to Dll4/Notch inhibitors [7,12].

Another ligand-receptor pair downstream from the VEGF and Notch pathways that plays a critical role in artery-vein endothelium specification is Ephrin-B2 and EphB4. Ephrin-B2 is specifically expressed in arterial angioblasts, endothelial cells, and perivascular mesenchymal cells, whereas EphB4 is expressed in endothelial cells belonging to the venous lineage only. Targeted disruption of either *EphB4 *or *EfnB2 *results in early lethality in the developing embryo as a result of arrested angiogenesis but not vasculogenesis [13-15].

On binding, the receptor and ligand on adjacent cells undergo dimerization and clusterization, activating forward and reverse signaling in receptor-expressing and ligand-expressing cells respectively to achieve vascular maturation. The monomeric form of the extracellular domain of EphB4 functions as an antagonist of EphB4-Ephrin-B2 signaling, thus blocking endothelial cell migration, tube formation and retards angiogenesis in tumor models [16,17]. Fusion of this protein with albumin at the C-terminus (sEphB4-Alb) results in favorable pharmokinetics for clinical development.

We chose to make use of the RIP1-Tag2 transgenic mouse model [18], wherein pancreatic islet carcinogenesis occurs secondary to the expression of the SV-40 large T-antigen (Tag) expression under the Rat Insulin Promoter (RIP). In this model, angiogenic islets become hyperplastic and dysplastic by week 5, and acquire angiogenic switch by week 10, progressing to adenomas (insulinomas) and invasive carcinomas [19]. Predictable stepwise progression and angiogenic switch permits investigation of tumor angiogenesis and their inhibitors.

This study was undertaken to test the activity of a Dll4/Notch inhibitor and an Ephrin-B2/EphB4 inhibitor, each alone and in combination. We studied the effects of *Dll4 *allelic deletion and of systemic administration of sDll4 on the tumor vasculature. In addition we studied the effect of sEphB4-Alb alone, in combination with sDll4 treatment or with *Dll4 *allelic deletion. The results validate the efficacy of each inhibitor alone and reveal for the first time that simultaneous inhibition of both pathways has greater efficacy. Furthermore, given the concerns related to potential toxicity caused by therapeutic blockade of Dll4 signaling [20], conditional Dll4 knockout mice were used to assess the impact of chronic endothelial specific *Dll4 *loss-of-function. This was observed to cause hepatic vascular alterations, as previously reported for pharmacological inhibition of Dll4/Notch signaling [20]. Interestingly, these lesions were prevented by systemic Ephrin-B2/EphB4 inhibition, which provides an important advantage to this combination therapy.

## Methods

### Experimental animals

All animal-involving procedures in this study were approved by the Faculty of Veterinary Medicine of Lisbon Ethics and Animal Welfare Committee. The generation of *Dll4^+/- ^*(*Dll4/LacZ*) mice on CD1 background has been reported previously [4]. The transgenic RIP1-Tag2 (RT2) mice of CD1 and C57/BL6 backgrounds, used for breeding with the *Dll4^+/- ^*line and in experimental drug trials, respectively, were provided by Dr. Oriol Casanovas. Dll4 conditional knockout mice (*Dll4^lox/lox^*) were generated as previously described [2] and crossed with VE-cadherin-Cre-ERT2 mice, a kind gift from Dr. Ralph Adams, to produce a tamoxifen-inducible endothelial-specific *Dll4 *loss-of-function line (*Dll4^lox/lox ^*Cre+). The animals were housed in well ventilated propylene cages with sawdust as bedding, in a room with controlled temperature between 22°C and 25°C and a 12-hours-light/12-hours-dark cycle. The mice were fed with standard laboratory diet and water *ad libitum*. From 12 weeks of age, all RT2 mice received 5% sugar in their water to relieve the hypoglycemia induced by the insulin-secreting tumors.

### Experimental design, tumor burden analyses and therapeutic trials

To study the effects of impaired Dll4/Notch signaling on RT2 insulinoma growth, RT2 *Dll4^+/+ ^*and RT2 *Dll4^+/- ^*littermates (CD1 background, n = 8 for each group) were sacrificed for tumor measurement, histological analysis of vascular morphology and gene expression analysis at 13.5 weeks of age. The pancreas glands were dissected and the macroscopic tumors (≥1 × 1 mm) were excised. Tumor volume was calculated using the formula *V *= 0.52 × *a *× *b*^2 ^where *a *and *b *equal the longer and shorter diameter of the tumor, respectively. The volumes of all tumors from each mouse were added to give the overall tumor burden per animal.

The effect of *Dll4 *allelic deletion in combination with Ephrin-B2/EphB4 signaling inhibition on the growth of the RT2 insulinoma was assessed by the administration of the soluble extracellular domain of EphB4 fused with albumin (sEphB4-Alb), which was produced as previously described [17]. Both RT2 Dll4^+/+ ^and RT2 Dll4^+/- ^mice (CD1 background, n = 12 for each group) were separated in equal subgroups, treated intraperitoneally (*i.p*.) with vehicle (PBS) or sEphB4-Alb (10 mg/kg) 3×/wk for 3.5 weeks beginning at the age of 10 weeks and finally sacrificed for tumor measurement and histological analysis.

In the therapeutical trials. we assessed the efficacy of a systemically administered Dll4/Notch-inhibitor, soluble Dll4 extracellular domain fused to Fc (sDll4), both alone and in combination with sEphB4-Alb. sDll4 was produced as previously described [8]. Vehicle (PBS, *i.p*. 3×/wk), sDll4 (10 mg/kg/day, *i.p*., 3×/wk), sEphB4-Alb (10 mg/kg/day, *i.p*., 3×/wk), and the combination of sDll4 (10 mg/kg/day, *i.p*., 3×/wk) with sEphB4-Alb (10 mg/kg/day, *i.p.*, 3×/wk) treatments were started when RT2 mice (C57BL6 background) reached the age of 10 weeks and continued until mice were 13.5 week-old. Two independent experiments involved 6 animals per treatment group.

### Longevity study

To evaluate the effect of *Dll4 *allelic deletion in combination with Ephrin-B2/EphB4 signaling inhibition on longevity, the RT2 *Dll4^+/+ ^*and RT2 *Dll4^+/- ^*mice were separated in two equal groups (n = 10 for each group), treated *i.p*. with vehicle (PBS) or sEphB4-Alb (5 mg/kg, 3×/wk), beginning at the age of 10 weeks and continuously monitored for signs of hypoglycemic shock. The mice were sacrificed if found moribund or if body weight loss exceeded 15%. Survival rate was calculated as the percentage of live mice at the end of each week relative to the initial number of animals in the experimental group.

### Assessment of toxicity

Heart, lung, liver, brain, kidney and intestines were collected from sDll4 and sEphB4-Alb treated mice used in the therapeutical trials, fixed in 10% formalin solution for 48 h, dehydrated in alcohol, cleared in xylene, embedded in paraffin, sectioned at 10 μm and stained with hematoxylin (Fluka AG Buchs SG Switzerland) and eosin Y (Sigma Chemicals, St. Louis, MO) to study eventual histopathological alterations. To assess the side effects that might arise from total (100%) inhibition of endothelial-specific Dll4 signaling, 8 week-old *Dll4^lox/lox ^*Cre+ (n = 10) were treated with tamoxifen (50 mg/kg daily for 5 days) to produce endothelial-specific Dll4 null individuals, while a group of 8 week-old *Dll4^lox/lox ^*Cre+ (n = 10) were left uninduced (control mice with constitutive *Dll4 *expression). Ten weeks later, the mice were sacrificed and heart, lung, liver, brain, kidney and intestines were collected, processed and examined as described above. Since *Dll4 *endothelial loss of function has been associated with hepatic lesions, we decided to determine the potential toxicological effect of a combination of Dll4/Notch and Ephrin-B2/EphB4 targeted therapy. Therefore another group of 8-week-old *Dll4^lox/lox ^*Cre+ (n = 10) were treated with tamoxifen (50 mg/kg daily) for 5 days, subsequently divided in two equal subgroups that were injected with vehicle (PBS) or sEphB4-Alb (10 mg/kg) for ten weeks and then sacrificed. Liver samples were processed and examined as described above.

### Immunohistochemistry

RT2 insulinomas obtained from tumor burden studies and therapeutical trials were fixed in a 4% paraformaldehyde (PFA) solution at 4°C for 1 h, cryoprotected in 15% sucrose, embedded in 7.5% gelatin, snap frozen in liquid nitrogen and cryosectioned at 10 and 20 μm. Double fluorescent immunostaining to the platelet endothelial cell adhesion molecule (PECAM) and the peri-vascular cell marker alpha smooth muscle actin (α-SMA) was performed on tissue sections to examine tumor vascular density and vessel maturity while double fluorescent immunostaining to PECAM and the pericyte marker neurogenin 2 chondroitin sulfate proteoglycan (NG2) was used to visualize pericyte recruitment. Rat monoclonal anti-mouse PECAM (BD Pharmingen, San Jose, CA), and rabbit polyclonal anti-mouse α-SMA (Abcam, Cambridge, UK) or rabbit polyclonal anti-mouse NG2 (Millipore, Billerica, MA) were used as primary antibodies. Species-specific secondary antibodies conjugated with Alexa Fluor 488 and 555 were from Invitrogen (Carlsbad, CA). Tissue sections were incubated with primary antibody overnight at 4°C and with secondary antibody for 1 hour at room temperature. Nuclei were counterstained with 4',6-diamidino-2-phenylindole dihydrochloride hydrate (DAPI; Molecular Probes, Eugene, OR). Fluorescent immunostained sections were examined under a Leica DMRA2 fluorescence microscope with Leica HC PL Fluotar 10 and 20X/0.5 NA dry objective, captured using Photometrics CoolSNAP HQ, (Photometrics, Friedland, Denmark), and processed with Metamorph 4.6-5 (Molecular Devices, Sunnyvale, CA). Morphometric analyses were performed using the NIH ImageJ 1.37 v program. Vessel density corresponds to the percentage of each tumor section field occupied by a PECAM-positive signal. As a measure of vascular maturity, vessel wall assembly was assessed by quantifying the percentage of PECAM-positive structures lined by α-SMA-positive coverage while pericyte recruitment was assessed by quantifying the percentage of PECAM-positive structures lined by NG2-positive coverage.

### Vessel perfusion study

To mark vessel perfusion, mice were anesthetized and biotin-conjugated lectin from *Lycopersicon esculentum *(100 μg in 100 μl of PBS; Sigma, St. Luis, MO) was injected via caudal vein and allowed to circulate for 5 minutes before the vasculature was transcardially perfused with 4% PFA in PBS for 3 minutes. Tumor samples were collected and processed as described above. Endothelial cells were stained with PECAM antibody and perfused vessels were visualised by streptavidin-Alexa 488 (Invitrogen, Carlsbad, CA), which binds to biotinylated lectin. The images were obtained and processed as described above. Tumor perfusion was quantified by determining the percentage of PECAM-positive structures that were colocalized with Alexa 488 signals.

### Global gene expression and quantitative transcriptional analysis

Tumors from sEphB4-Alb or PBS treated RT2 *Dll4^+/+ ^*mice were harvested at week 13.5. RNA was then isolated and used for global gene expression analysis with Illumina MouseRef-8 v2.0 Expression BeadChip (Illumina, San Diego, CA). The genearray data were deposited to NCBI-GEO database http://www.ncbi.nlm.nih.gov/geo/query/acc.cgi?acc=GSE24603. Genes with expression change between two groups higher than 2 fold and P value smaller than 0.05 were selected and the changes were validated by quantitative RT-PCR.

Using a SuperScript III FirstStrand Synthesis Supermix (Invitrogen, Carlsbad, CA), first-strand cDNA was synthesized from total RT2 *Dll4^+/+ ^*and RT2 *Dll4^+/- ^*insulinoma RNA. Real-time PCR analysis was performed as described [4] using specific primers for *β-actin, GAPDH, PECAM, Dll4, Hey2, VEGF-A, VEGFR1, VEGFR2, VEGF-C, VEGFR3, PDGF-β, Ephrin-B2, and Tie2*. Primer pair sequences are available on request. Gene expression was normalized to *β-actin and GAPDH*.

### Statistical analyses

Data processing was carried out using the Statistical Package for the Social Sciences version 15.0 (SPSS v. 15.0; Chicago, IL). Kaplan-Meier product-limit estimation with Breslow generalized Wilcoxon test was used for survival analyses. All other statistical analyses were performed using the Mann-Whitney-Wilcoxon test. All results are presented as mean ± SEM or mean ± SD when more appropriate. *P*-values < 0.05 and <0.01 were considered significant (indicated in the figures with *) and highly significant (indicated with **), respectively.

## Results

### Targeted *Dll4 *allele deletion reduces tumor growth in RT2 mice

**Figure 1:**
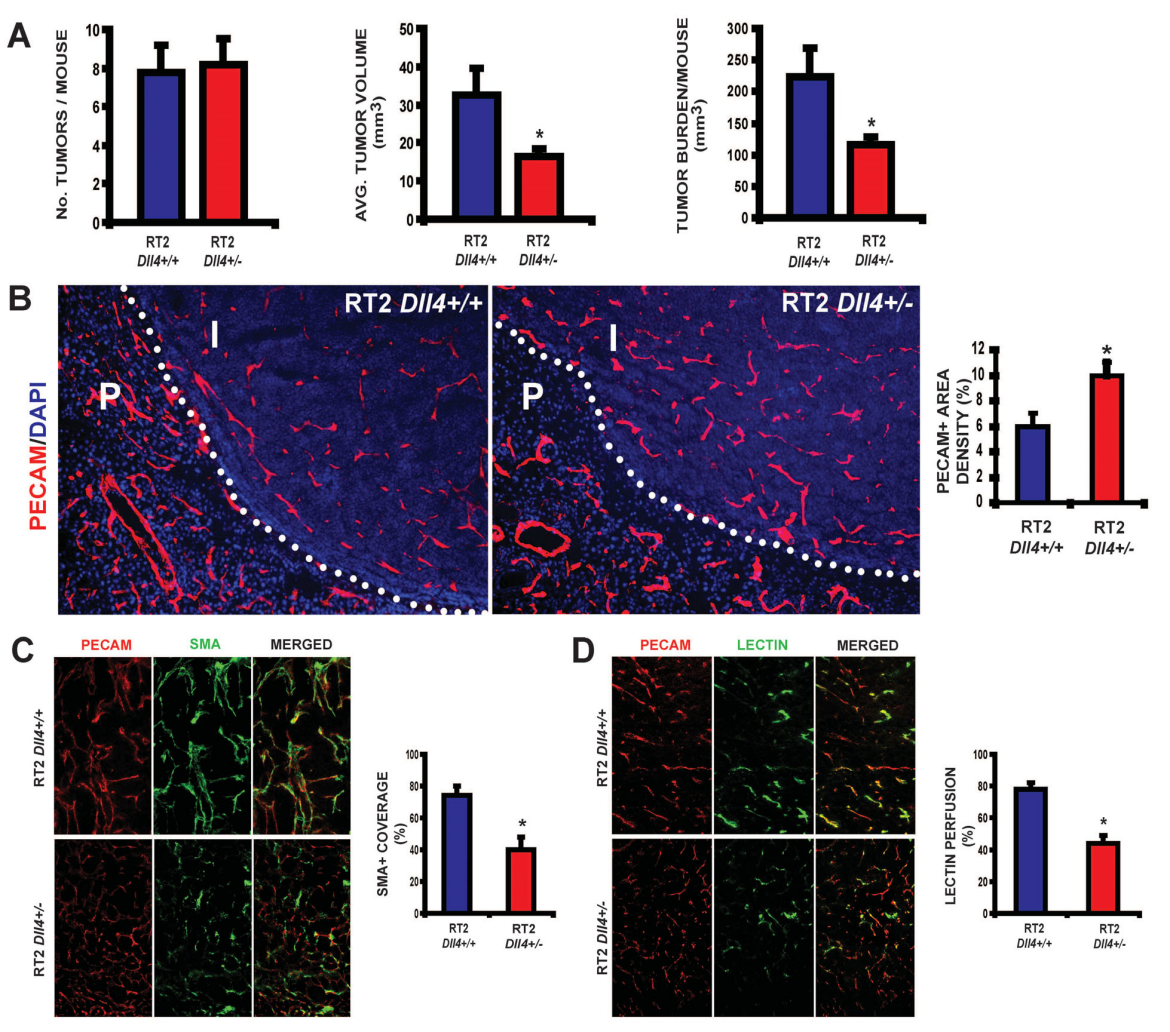
***Dll4 *allelic deletion reduced tumor burden in RT2 mice due to increased nonproductive tumor angiogenesis**. *A*, Number of tumors per animal, average tumor volume and overall tumor burden, are calculated as the sum of tumor volumes per mouse, in RT2 *Dll4^+/+ ^*(n = 8) and RT2 *Dll4^+/- ^*(n = 8) mice. *B*, Vascular response examined in RT2 *Dll4^+/+ ^*(n = 5) and RT2 *Dll4^+/- ^*(n = 5) insulinomas by PECAM immunostaining. RT2 *Dll4^+/- ^*mice showed reduced vessel calibers, increased sprouting, branching irregularities, and network disorganization (*left*). Dotted lines mark tumor border. *I *indicates insulinoma while *P *indicates normal pancreatic tissue surrounding the tumor. Vascular density was estimated as percentage of PECAM-positive area per tumor section surface and presented for two experimental groups (*right*). *C*, Mural cell coverage on newly formed vessels was examined by double staining of PECAM and α-SMA. SMA-positive cells lining PECAM-positive endothelium were profoundly reduced in RT2 *Dll4^+/- ^*insulinomas (*left*). The percentage of PECAM-positive structures covered by SMA-positive area was measured as an indicator of vessel maturation (*right*). *D*, Tumor vessel competence evaluated by lectin perfusion. Simultaneous staining of endothelial cells with PECAM and visualization of perfused vessels demonstrate a reduction in the fraction of perfused vessels in RT2 *Dll4+/- *vs. RT2 *Dll4+/+ *mice (*left*). The percentage of PECAM-positive area co-localized with lectin was measured to estimate the proportion of functional vessels within insulinomas (*right*). The two group values for each parameter were statistically analyzed using Mann-Whitney-Wilcoxon test. *Error bars *represent SEM. * *P *< 0.05 was considered significant.

To assess the effect of impaired signaling through the Dll4/Notch pathway in an autochthonous tumor model, we crossed RT2 transgenic mice with *Dll4^+/- ^*mice and compared tumor burden between RT2 *Dll4^+/+ ^*and RT2 *Dll4^+/- ^*offspring. The two groups were humanely sacrificed at 13.5 weeks of age. Average tumor numbers per mouse were similar in RT2 *Dll4^+/+ ^*and RT2 *Dll4^+/- ^*littermates (Figure 1A). In contrast, a significant decrease in tumor growth was observed in RT2 *Dll4^+/- ^*mice when compared to the RT2 *Dll4^+/+ ^*control group. On average, RT2 *Dll4^+/- ^*insulinoma volume and overall tumor burden, calculated as a sum of tumor volumes per mouse, were reduced by approximately 50% compared to the control animals (Figure 1A).

### Partial Dll4/Notch suppression due to *Dll4 *allelic deletion promotes immature and non-functional vessel proliferation in RT2 insulinomas

We also examined the vascular morphology of tumors derived from both RT2 *Dll4^+/+ ^*and RT2 *Dll4^+/- ^*mice at week 13.5. Tumor endothelium was visualized by immunostaining of PECAM while PECAM/α-SMA co-localization was used to evaluate mural cell recruitment and thereby vessel maturity. In addition, subgroups of RT2 *Dll4^+/+ ^*and RT2 *Dll4^+/- ^*mice were perfused with endothelium-binding lectin to visualize the perfused regions of the tumor vasculature. The RT2 *Dll4^+/+ ^*tumors had irregular vessel formation and absence of a distinct branching pattern (Figure 1B) as well as a high degree of vessel maturity with a mean α-SMA-positive cell coverage of approximately 75% (Figure 1C). Furthermore, the lectin-perfusion study indicated that nearly 80% (78 ± 4%) of the vessels displayed appropriate lumen formation and were perfused (Figure 1D). In comparison, RT2 *Dll4^+/- ^*insulinoma showed a 1.8-fold increase in the density of PECAM-positive areas (p < 0.05) and formed atypical networks lacking vessel hierarchy or symmetry but displaying pronounced branching and multiple interconnections (Figure 1B). The number of α-SMA-positive cells lining PECAM-positive endothelial cells was greatly reduced (45% reduction, p < 0.05, Figure 1C). As anticipated, a significant reduction in the proportion of functional vessels was demonstrated by lectin-perfusion (2-fold decrease, p < 0.05, Figure 1D).

### Reduced Dll4/Notch signaling in the RT2 tumor affects expression of vessel growth regulators and/or their receptors

**Figure 2:**
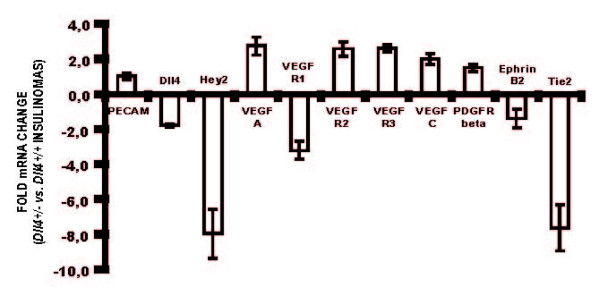
**Differential gene expression in RT2 *Dll4*^+/+ ^vs. RT2 *Dll4^+/- ^*insulinomas**. Total RNA was isolated from harvested insulinomas (2-3 tumors/mouse, n = 5 for each genotype) and gene expression analysis of tumor tissues was performed by quantitative RT-PCR for indicated genes involved in angiogenesis. Gene expression levels were normalized to *β-actin *levels. Error bars represent SD.

Quantitative RT-PCR was used to analyze RT2 *Dll4^+/+ ^*and RT2 *Dll4^+/- ^*tumors for putative differences in the expression of genes known to be involved in physiological and tumor angiogenesis (Figure 2). Relative to *Dll4^+/+ ^*insulinomas, *Dll4^+/- ^*tumors showed an approximately 50% reduction in *Dll4 *mRNA levels, as expected, as well as reduced *Hey2 *expression, consistent with a reduction in Notch signaling. Furthermore, there was increased *Vegf-a *expression and an over 8-fold increase in the VEGFR2/VEGFR1 ratio in the *Dll4^+/- ^*tumors. Similarly, both *Vegf-c *and *Vegfr3 *were up-regulated in these tumors. In addition, there was an increase in *PDGFR-β*, and decrease in *Ephrin-B2 *and *Tie2 mRNA levels*. The RNA level of PECAM was not significantly increased (1.2 fold), different from 40% increase of the PECAM signal in Figure 1B. One explanation is that quantitative PCR was done in whole tumor tissue and not microdissected vessels, which likely resulted in failure to document PECAM modulation in mRNA level.

### sEphB4-Alb potentiates the effects of *Dll4 *allelic deletion on RT2 insulinoma growth and survival rate by further antagonizing tumor vessel maturation

**Figure 3:**
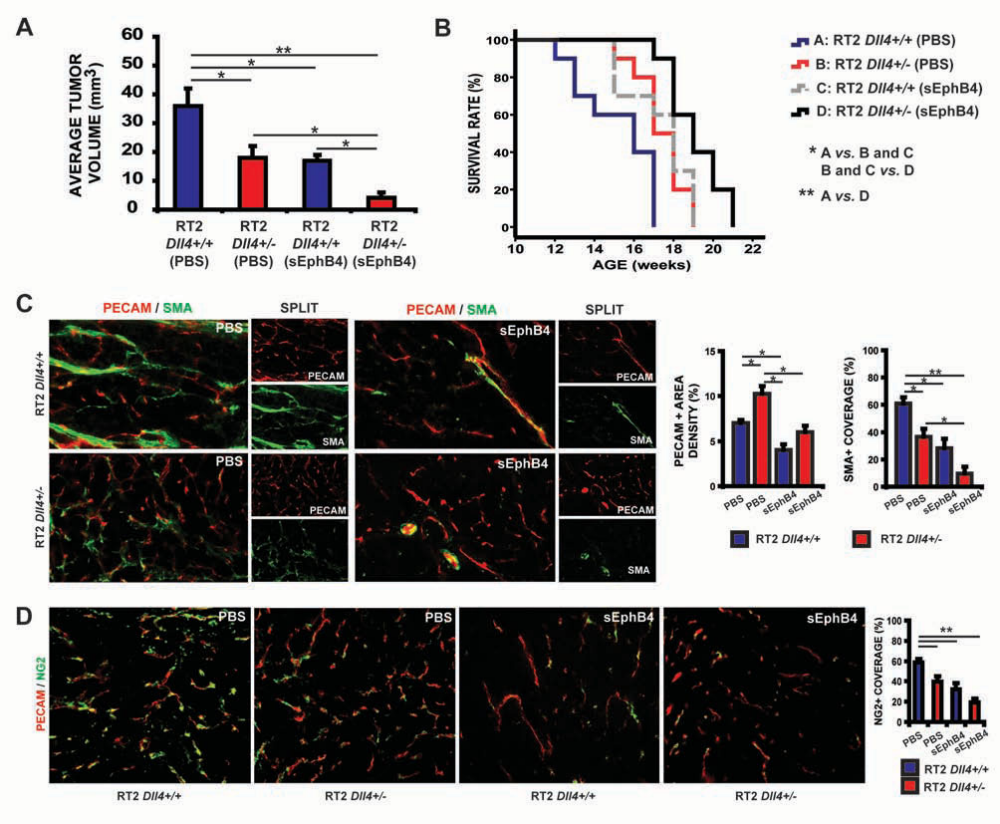
**sEphB4-Alb inhibited insulinoma growth and extended longevity in RT2 *Dll4^+/+ ^*and *Dll4^+/- ^*mice**. *A*, Average tumor volumes developed by 13.5-week old RT2 (*Dll4^+/+ ^*or *Dll4^+/-^*) male mice previously treated for 3.5 weeks with drug vehicle (PBS) or sEphB4-Alb (10 mg/kg). All the groups had 6 animals and were treated i.p. 3 times a week. *B*, Survival rates in RT2 *Dll4^+/+ ^*and RT2 *Dll4^+/- ^*treated with vehicle (PBS) or sEphB4-Alb (5 mg/kg). All the groups had 10 animals and were treated i.p. 3 times a week. Survival rate was calculated as the percentage of live mice at the end of each week relative to the initial number of the animals per experimental group. *C*, Sections of tumors harvested at the end of the experiment mentioned in *A *were co-stained with anti-PECAM (*red*) and anti-α-SMA (*green*) as described in Fig. 1*C*. *D*, Sections of tumors harvested at the end of the experiment mentioned in *A were *co-stained with anti-PECAM (*red*) and anti-NG2 (*green*). The percentage of PECAM-positive structures covered by NG2-positive area was measured as a parameter of pericyte recruitment (*right*). Kaplan-Meier product-limit estimation with Breslow generalized Wilcoxon test was used for survival analyses. Other data were analyzed using Mann-Whitney-Wilcoxon test. *Error bars *represent SEM. * *P *< 0.05 and ** *P *< 0.01 were considered significant and highly significant, respectively.

sEphB4-Alb has been shown to be a potent inhibitor of tumor angiogenesis by blocking Ephrin-B2 and EphB4 signaling. To examine the effect of sEphB4-Alb in the RT2 system, RT2 mice were treated with sEphB4-Alb or PBS for 3.5 weeks beginning at 10 weeks of age. At 13.5 weeks of age, sEphB4-Alb treated RT2 mice showed reduced mean tumor volumes relative to PBS-treated control mice by approximately 50% (p < 0.05) (Figure 3A). At this point tumors were harvested and RNA was isolated and used for global gene expression analysis. Genes with expression changes between the two groups higher than 2-fold and P value smaller than 0.05 are shown in Table 1. Selected genes with known role in vascular biology were evaluated by quantitative RT-PCR for validation studies. Of these genes, *Rgs5 *and *Psenen *are of special interest. They are upregulated in sEphB4-Alb treated group compared to the untreated wild type mice and their changes were validated by quantitative RT-PCR (supplementary Figure 1A). Rgs5 is a marker of pericytes and is a negative regulator of pericyte maturation. Rgs5 was also upregulated in umbilical artery smooth muscle cell co-cultured with human umbilical artery endothelial cell when treated with sEphB4-Alb (Additional file 1. Psenen is a critical component of presenilin complex that is required for Notch receptor processing.

**Table 1:** Upregulated genes in sEphB4-Alb treated RT2 mice

Gene name	Fold Change	P value
9430028L06Rik	2.11	0.02

Acta2	2.26	0.05

Akr1b8	2.26	0.02

Anpep	2.10	0.03

Atp6v1a	2.58	0.05

BC048546	2.16	0.01

Bgn	3.06	0.05

Bmp4	2.21	0.03

C3	2.58	0.05

C4b	2.17	0.02

Ccdc32	2.44	0.01

Cd52	2.90	0.04

Cfd	2.79	0.02

Clec4n	2.30	0.03

Cox7a1	2.63	0.01

Cyba	2.46	0.01

D430039N05Rik	2.66	0.04

Dab2	2.02	0.05

Foxp1	2.24	0.05

Fus	2.42	0.01

Gde1	2.20	0.04

H2-DMb1	2.21	0.04

Hoxb2	2.40	0.04

Il3ra	2.00	0.05

Ins1	3.00	0.01

Iqgap1	2.29	0.05

Lamp2	2.55	0.02

Lgals1	2.48	0.02

Ms4a6d	2.23	0.04

Ncf4	2.10	0.04

Ndrg4	2.13	0.04

Nmu	2.37	0.03

P2ry6	2.45	0.04

Pdia3	2.21	0.03

Ppp2r4	2.05	0.03

Prelp	2.48	0.05

Prph	2.89	0.01

Psenen	2.18	0.02

Pyy	3.44	0.01

Rbp1	2.96	0.02

Rgs5	2.66	0.01

Rnasek	2.03	0.03

Samd9l	2.45	0.05

Scara3	2.33	0.05

Slc15a3	2.12	0.04

Slc1a3	2.06	0.00

St3gal6	2.46	0.04

Tax1bp3	3.20	0.04

Usp11	2.00	0.03

Zcchc12	2.03	0.04

Zfp36l1	2.06	0.04

To examine whether synchronous suppression of Dll4/Notch and EphB4/Ephrin-B2 signaling pathways might result in greater inhibition of RT2 tumor development, we treated RT2 *Dll4^+/- ^*mice with sEphB4-Alb. The reduction of the average tumor volume was even more pronounced in sEphB4-Alb treated RT2 *Dll4^+/- ^*group (approximately 90% reduction, p < 0.01) where Dll4/Notch and Ephrin-B2/EphB4 signaling were simultaneously inhibited (Figure 3A).

To test whether these tumor-suppressive effects are reflected on longevity, both *Dll4^+/+ ^*and *Dll4^+/- ^*RT2 mice were treated with PBS or half dose of sEphB4-Alb (5 mg/kg) beginning at 10 weeks of age and continuously monitored for survival benefit estimation. *Dll4 *allelic deletion just as sEphB4-Alb treatment were found to extend the mean lifespan of RT2 mice by 2 weeks (15 ± 1 wk in PBS treated RT2 *Dll4^+/+ ^versus *17 ± 1 wk in PBS treated RT2 *Dll4^+/- ^*and sEphB4-Alb treated RT2 *Dll4^+/+ ^*mice, p < 0.05 Figure 3B). Significantly, an additional survival benefit of 2 weeks, on average, was provided by combinatorial Dll4/Notch and Ephrin-B2/EphB4 inhibition (15 ± 1 wk in PBS-treated RT2 *Dll4^+/+ ^versus *19 ± 1 wk in sEphB4-treated RT2 *Dll4^+/-^*, p < 0.01, Figure 3B).

Histologicaly, microvessel density assessed by immunostaining of PECAM was significantly increased in PBS treated *Dll4^+/- ^*tumors compared to PBS treated *Dll4^+/+ ^*insulinomas, while sEphB4-Alb treatment in RT2 *Dll4^+/+ ^*mice caused a statistically significant decrease of tumor vessel density (Figure 3C). Notably, in both PBS treated *Dll4^+/- ^*tumors and sEphB4-Alb treated RT2 *Dll4^+/+ ^*tumors, average vessel caliber was reduced, and pericyte recruitment and vessel wall formation were markedly impaired, as indicated by PECAM/NG2 and PECAM/α-SMA immunostaining (Figure 3C and 3D). Furthermore, the simultaneous suppression of Dll4/Notch and Ephrin-B2/EphB4 signaling in sEphB4-Alb treated RT2 *Dll4^+/- ^*mice drastically reduced the vessel diameters and their mural cell coverage, even though the total PECAM-positive area apparently similar to PBS-treated *Dll4^+/+ ^*insulinomas (Figure 3C and 3D). Therefore, combinational targeting Dll4/Notch signaling EphrinB2-EphB4 has greater efficacy in blocking vessel maturation and perfusion of the tumor.

### Both sDll4 and sEphB4-Alb inhibit RT2 tumor growth and act in a cumulative manner

**Figure 4:**
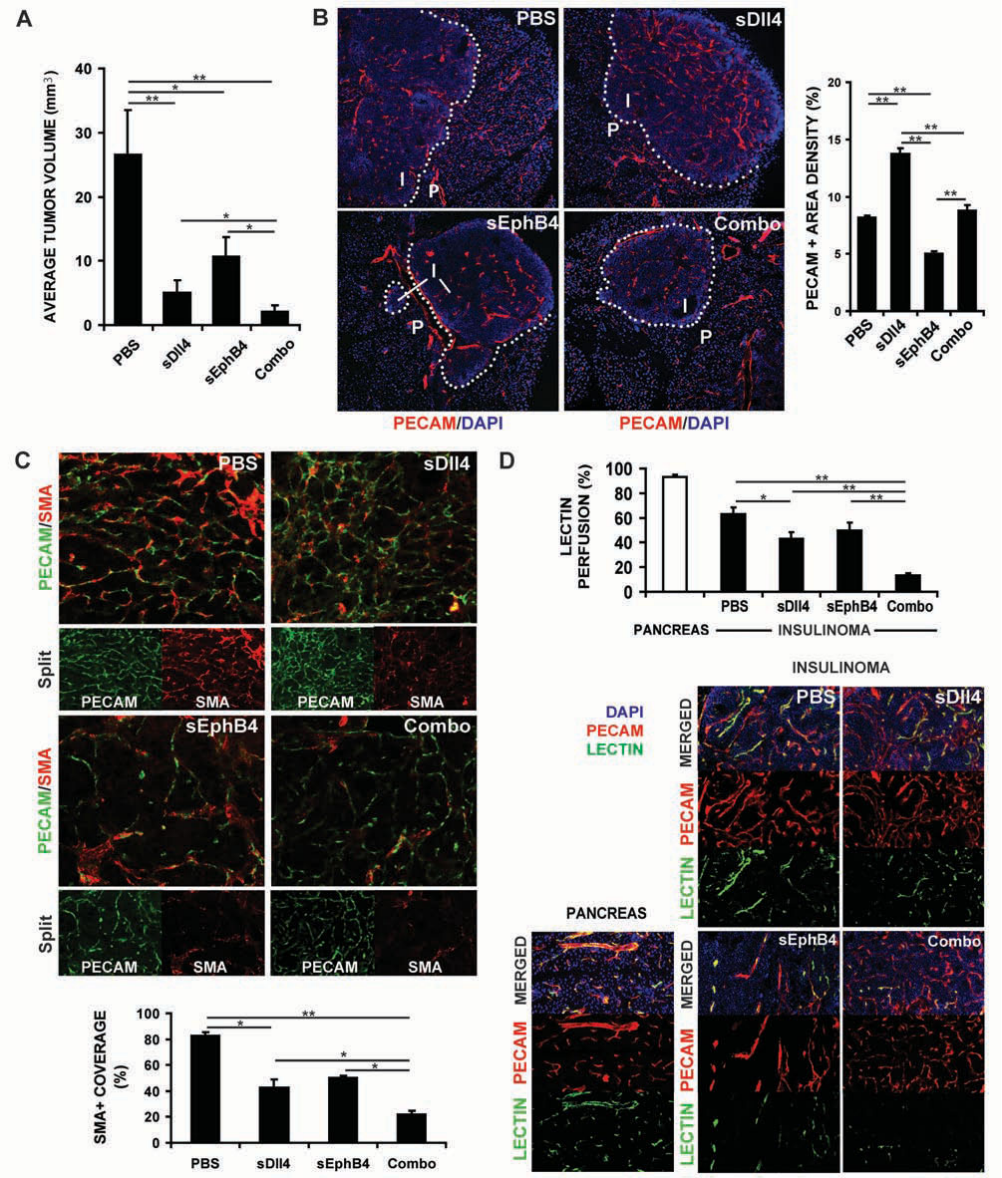
**Combination therapy of sDll4-Fc and sEphB4Alb inhibits RT2 tumor growth with greater efficacy than either molecule alone**. *A*, Average tumor volumes developed by 13.5-week old RT2 male mice previously treated for 3.5 weeks with drug vehicle (PBS, i.p. 3×/wk, control group), sEphB4-Alb (10 mg/kg i.p., 3×/wk), sDll4-Fc (10 mg/kg i.p. 3×/wk), or combination of sEphB4-Alb and sDll4-Fc (10 mg/kg, i.p. 3×/wk for both). The results were obtained from two independent trials involved 6 animals per treatment group and per trial. *B*, Sections of tumors harvested at the end of the experiment mentioned in *A *were immunostained with PECAM (*red*) and DAPI (*blue*) as described in Fig. 1*B*. *C*. Sections of tumors harvested at the end of the experiment mentioned in *A *were stained as described in Fig. 1*C*. *D*, Tumor vessel functionality evaluated by lectin perfusion as described in Fig. 1*D*. Perfusion in normal pancreas tissue was also analyzed and shown on the bottom left. The data were analyzed using Mann-Whitney-Wilcoxon test. Error bars represent SEM. * *P *< 0.05 and ** *P *< 0.01 were considered significant and highly significant, respectively.

sDll4 is a potent inhibitor of Notch signaling and has been shown to reduce tumor growth in various tumor models [7,8,21]. We sought to determine the effect of combined blockade of Dll4/Notch pathway (by sDll4) and Ephrin-B2/EphB4 pathway (by sEphB4-Alb) on tumor angiogenesis. RT2 mice were treated from 10 to 13.5 weeks of age. Compared to control mice treated with the vehicle (PBS), tumor volumes were reduced in both sDll4 treated (81% reduction, p < 0.01) and sEphB4-Alb treated animals (60%, p < 0.05). As presented in Figure 4A, the combination therapy caused an even more significant tumor suppression (92%, p < 0.01), which has significant difference when compared to the therapy with sDll4 or sEphB4-Alb alone (p < 0.05).

Harvested tumors were double immunostained for PECAM/α-SMA to analyze microvessel density and maturity and PECAM/lectin to estimate tumor perfusion and vessel functionality (Figure 4B-D). Compared to PBS treated group, sDll4 treated tumors showed an increase in total tumor vessel density of 41% on average (p < 0.01), reduced mural cell coverage of 47% (p < 0.05), and reduced lectin perfusion of 32% (p < 0.05) sEphB4-Alb treatment in contrast caused reduced microvessel density (39% reduction, p < 0.01), pericyte recruitment (39%) and lectin perfusion (21%). Finally, the small sized tumors observed in mice treated with both sDll4 and sEphB4-Alb were characterized by decreased vessel diameters (Figure 4B) and markedly impaired vessel maturation with 73% reduction in mural cell coverage compared to PBS-treated insulinomas (p < 0.01) (Figure 4C). Meanwhile, lectin perfusion was drastically decreased (79%, p < 0.01) (Figure 4D). These results indicate that sDll4/sEphB4-Alb combination therapy has at least a cumulative effect on minimizing tumor vessel competency and blood delivery to neoplasic cells, consequently reducing tumor growth.

### Endothelial Dll4/Notch inhibition results in hepatic vascular alterations that can be prevented by concomitant inhibition of Ephrin-B2/EphB4

**Figure 5:**
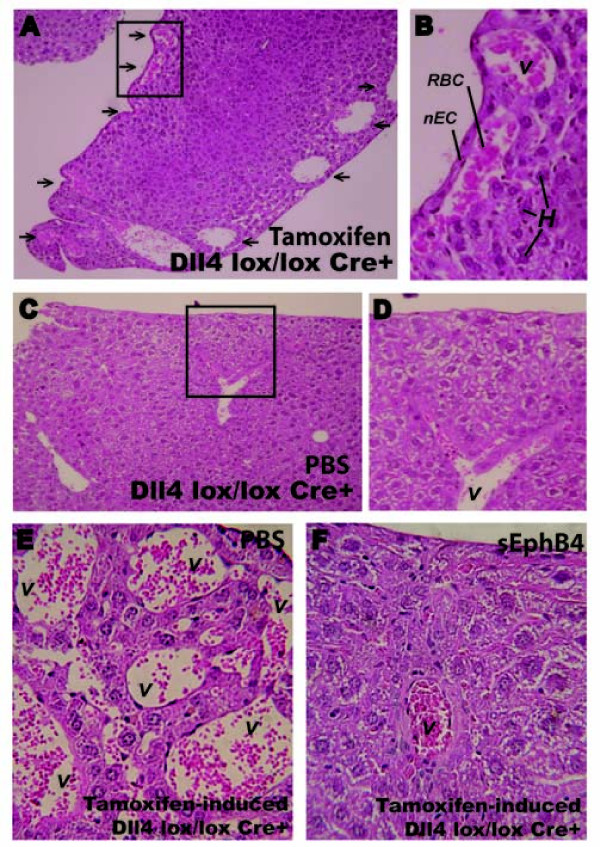
**Hepatic vascular lesions observed in endothelial-specific *Dll4 *knock-out mice are prevented by sEphB4-Alb administration**. Representative pictures of hematoxylin and eosin staining of liver sections from induced, tamoxifen-treated (*A *and *B*) and non-induced, PBS-treated (*C *and *D*) *Dll4^lox/lox ^Cre+ *mice, and from induced, tamoxifen-treated *Dll4^lox/lox ^Cre+ *mice administered with PBS vehicle (*E*) or sEphB4-Alb (*F*) for 12 weeks to assess the effect of Ephrin-B2/EphB4 blockade combined with total inhibition of Dll4/Notch endothelial signaling. *A*, Ten weeks after the induction of endothelium-specific Dll4 loss-of-function, all studied 14-week-old *Dll4^lox/lox ^*Cre+ presented excessive sub-capsular vascular proliferation. The image shows vessels (*arrows*), some of which extremely dilated, occupying sub-capsular regions throughout the liver. *B*, Higher magnification of sub-capsular blood vessels (inset in *A*); *C*, Age-matched non-induced *Dll4^lox/lox ^*Cre+ presented normal liver histology. *D*, Higher magnification of the inset in *C *showing a single hepatic vessel that cannot be considered sub-capsular. *E *and *F*, Excessive vascular proliferation forming an hemangioma-like sub-capsular structure in a tamoxifen-induced *Dll4^lox/lox ^Cre+ *mouse injected with PBS and normal hepatic structure in a sEphB4-treated tamoxifen-induced *Dll4^lox/lox ^Cre+ *mouse, respectively. *V*, blood vessel; *nEC*, endothelial cell nucleus, *RBC*, red blood cells; *H*, hepatocytes.

Chronic Dll4 blockade results in benign vascular proliferative lesions in the liver [4]. In contrast, *Dll4 *haploinsufficiency and intermittent administration of sDll4 (3 times a week for 3.5 weeks) in RT2 mice significantly reduced tumor growth, but did not cause any vascular lesions in the liver or other vital organs such as heart, brain, lung, kidney, and intestine (data not shown). To confirm if complete and persistent loss of Dll4 causes organ toxicity, we used a conditional, endothelial-specific *Dll4 *knock-out mouse line (*Dll4^lox/lox ^*Cre+), in which *Dll4 *deletion is dependent on tamoxifen administration. Comparative histological analyses were performed with cardiac, cerebral, pulmonary, renal, intestinal and hepatic samples obtained from mice 10 weeks after tamoxifen-induction or vehicle (PBS)-administration. While control animals presented normal organ histology, the endothelial Dll4 knock-out mice showed alterations in liver architecture. Macroscopically, the liver surface of tamoxifen-induced Dll4^lox/lox ^Cre+ mice had a characteristic micro-nodule. Although general lobular architecture and portal spaces were preserved, we observed some areas with markedly dilated sinusoids. However, the most prominent feature was excessive subcapsular vessel proliferation with a few hemangioma-like structures (Figure 5A and 5B). To assess whether and to which extent these alterations can be influenced by concomitant Ephrin-B2/EphB4 inhibition, we treated tamoxifen-induced Dll4^lox/lox ^Cre+ mice with PBS or sEphB4-Alb (10 mg/kg). While PBS treated mice developed previously described subcapsular vascular alterations (Figure 5E), sEphB4-Alb treated animals showed a few dilated sinusoids but no evidence of vascular proliferative lesions (Figure 5F).

## Discussion

*Dll4 *allelic deletion or sDll4 treatment reduces the rate of tumor growth in this spontaneous model of insulinoma in the pancreas, while the number of tumors per mouse were unchanged, indicating that the transforming event caused by SV40 large T antigen under insulin promoter is independent on Dll4/Notch pathway, while the tumor growth is promoted by the expression of Dll4 in the vasculature. Similarly, sEphB4-Alb, which blocks Ephrin-B2 mediated forward signaling in venous endothelial cells through its cognate receptors, in particular EphB4, and reverse signaling in the arterial endothelial cells expressing Ephrin-B2, reduces the rate of tumor growth without affecting the number of oncogenic tumor nodules in the pancreas. Combination of sEphB4-Alb and sDll4 had a greater tumor inhibition efficacy than each one alone. This novel finding indicates that the activity of both pathways is non-overlapping even though Dll4/Notch signaling induces Ephrin-B2 [22-24]. The lack of overlapping effects is consistent with the fact that Dll4 targeted therapy has a markedly increased number of tumor vessels while sEphB4-Alb therapy results in significantly reduced tumor vessel density. Inhibition of Dll4-Notch signaling leads to induction of VEGF, probably through hypoxia. Meanwhile, there is also an increase in VEGFR2 level, resulting in a positive loop leading to enhanced VEGF signaling and thus increased vascular density. In addition, VEGFR3 is induced in insulinomas of *Dll4^+/- ^*RT2 mice compared to the *Dll4^+/+ ^*RT2 littermates. VEGFR3 expression in nascent vessels in addition to lymphatic endothelium may thus also have contributed to the enhanced vascular response. sEphB4-Alb mediated inhibition of Ephrin-B2/EphB4 signaling also results in areas of hypoxia in the tumor and increased expression of VEGF and Dll4 [17], which support the combined use of Ephrin-B2, VEGF, and Dll4 inhibitors. However, sEphB4-Alb treatment leads to fewer tumor blood vessels, suggesting that inhibition of Ephrin-B2/EphB4 pathway may reduce VEGF signaling even though VEGF level is increased. This can be explained by the recent findings that Ephrin-B2 controls internalization and signaling of VEGFR2 and VEGFR3 [25,26]. VEGF receptor expression and internalization (trafficking), rather than the VEGF level, determine the net endothelial response. Concomitant inhibition of these two signaling systems results in vessel density below the controls, suggesting that reduction of VEGF signaling by Ephrin-B2/EphB4 inhibition partially overcomes the VEGF/VEGFR2 activation resulting from Dll4/Notch blockade. More importantly greater anti-tumor efficacy of combined therapy may result from markedly reduced vessel perfusion and maturation.

Interestingly, sEphB4-Alb treatment induces the expression of PSENEN, a critical component of gamma-secretase presenilin complex [27]. Presenilin complex is responsible for the cleavage of Notch receptor to release Notch intracellular domain, thus is required for activation of Notch signaling [28]. Significance of PSENEN modulation in response to sEphB4-Alb is not known at present.

In *Dll4^+/- ^*mice or sDll4 treated mice, the defects in vessel maturation may be in part be explained by the impaired Notch3 signaling, which is critical for mural cell function [29,30]. Moreover, one critical step of vessel maturation, the recruitment of mural cells to the newly forming vessels, is known to be regulated by Ephrin-B2, PDGFRβ/PDGF-B, Tie-2, and Sphingosine-1 phosphate [31-34]. We thus examined these factors after Dll4/Notch inhibition. Surprisingly, increased PDGFR-β levels were identified in RT2 *Dll4^+/- ^*vs. RT2 *Dll4^+/+ ^*insulinomas even though there was a prominent reduction in mural cell recruitment to the vessels. We believe that defective recruitment results in a feedback increase in PDGFR-β. Angiopoietin receptor Tie2 [35,36] as expected was decreased in Dll4^+/- ^compared to Dll4^+/+ ^insulinomas. The impaired mural cell recruitment upon Dll4/Notch inhibition may also be explained by the reduced Ephrin-B2 level. Ephrin-B2 is critical for the recruitment of mural cells to nascent vessels and its absence leads to vessel dilation and bleeding [14,37]. In this study, sEphB4-Alb significantly impaired the recruitment of mural cells to blood vessels. The global gene expression analysis identified Rgs5 is upregulated upon sEphB4-Alb treatment both in vivo and in co-cultured smooth muscle cells and endothelial cells. Rgs5 is a pericyte specific marker. Knockout of Rgs5 in mice promotes the maturation of pericytes and normalization of tumor vessels [38]. Rgs5 is also a novel HIF-1-dependent, hypoxia-induced gene that is involved in the induction of endothelial apoptosis [39]. Therefore, upregulation of Rgs5 is consistent with the decreased vascular density and paucity of pericyte recruitment after sEphB4-Alb treatment which results in immature and non-functional vessels.

Finally, in addition to improved anti-tumor efficacy, simultaneous blockade of Dll4/Notch and Ephrin-B2/EphB4 abolished the liver vascular lesions seen when only Dll4/Notch is inhibited [20]. sEphB4-Alb blocks this toxicity, consistent with the reduced vascular response upon Ephrin-B2/EphB4 inhibition. Thus, Ephrin-B2/EphB4 blockade combined with Dll4/Notch inhibition provides greater safety and improved efficacy.

## Conclusion

In summary, targeting of Dll4/Notch and Ephrin-B2/EphB4 in combination showed marked improvement in tumor growth inhibition. This is primarily through modulation of vascular response in which impaired vessel maturation leads to poor perfusion. Complementary to improved efficacy, combination of sEphB4-Alb with Dll4/Notch inhibition appears to prevent excessive vascular proliferation in the liver, which is seen in extended Dll4/Notch blockade alone. Therefore, this combination is a potential candidate for cancer therapy.

## Competing interests

The authors declare that they have no competing interests.

## Authors' contributions

DD, JG, MB, and AT carried out the animal studies and immunostaining. XL performed analysis of gene array. MG carried out the validation of gene array results. LS performed liver toxicity analysis. VK and RL produced the therapeutic proteins for animal studies. AD and PSG designed and coordinated the studies. DD, AD, PSG, and RL prepared the manuscript. All authors read and approved the final manuscript.

## Pre-publication history

The pre-publication history for this paper can be accessed here:

http://www.biomedcentral.com/1471-2407/10/641/prepub

## Supplementary Material

Additional file 1 **Upregulation of Rgs5 and PSENEN by sEphB4-Alb**. Upregulation of Rgs5 and PSENEN in sEphB4-Alb treated RT2 mice was confirmed by quantitative RT-PCR. Regulation of Rgs5 by sEphB4-Alb was also investigated in smooth muscle cells co-cultured with endothelial cells. Click here for file

## References

[B1] Loges S Roncal C Carmeliet P Development of targeted angiogenic medicine J Thromb Haemost 2009 7 1 21 33 10.1111/j.1538-7836.2008.03203.x 18983480

[B2] Bergers G Hanahan D Modes of resistance to anti-angiogenic therapy Nat Rev Cancer 2008 8 8 592 603 10.1038/nrc2442 18650835 PMC2874834

[B3] Gale NW Dominguez MG Noguera I Pan L Hughes V Valenzuela DM Murphy AJ Adams NC Lin HC Holash J Thurston G Yancopoulos GD Haploinsufficiency of delta-like 4 ligand results in embryonic lethality due to major defects in arterial and vascular development Proc Natl Acad Sci USA 2004 101 45 15949 54 10.1073/pnas.0407290101 15520367 PMC524697

[B4] Duarte A Hirashima M Benedito R Trindade A Diniz P Bekman E Costa L Henrique D Rossant J Dosage-sensitive requirement for mouse Dll4 in artery development Genes Dev 2004 18 20 2474 8 10.1101/gad.1239004 15466159 PMC529534

[B5] Patel NS Li JL Generali D Poulsom R Cranston DW Harris AL Up-regulation of delta-like 4 ligand in human tumor vasculature and the role of basal expression in endothelial cell function Cancer Res 2005 65 19 8690 7 10.1158/0008-5472.CAN-05-1208 16204037

[B6] Mailhos C Modlich U Lewis J Harris A Bicknell R Ish-Horowicz D Delta4, an endothelial specific notch ligand expressed at sites of physiological and tumor angiogenesis Differentiation 2001 69 2-3 135 44 10.1046/j.1432-0436.2001.690207.x 11798067

[B7] Noguera-Troise I Daly C Papadopoulos NJ Coetzee S Boland P Gale NW Lin HC Yancopoulos GD Thurston G Blockade of Dll4 inhibits tumour growth by promoting non-productive angiogenesis Nature 2006 444 7122 1032 7 10.1038/nature05355 17183313

[B8] Scehnet JS Jiang W Kumar SR Krasnoperov V Trindade A Benedito R Djokovic D Borges C Ley EJ Duarte A Gill PS Inhibition of Dll4-mediated signaling induces proliferation of immature vessels and results in poor tissue perfusion Blood 2007 109 11 4753 60 10.1182/blood-2006-12-063933 17311993 PMC1885521

[B9] Williams CK Li JL Murga M Harris AL Tosato G Up-regulation of the Notch ligand Delta-like 4 inhibits VEGF-induced endothelial cell function Blood 2006 107 3 931 9 10.1182/blood-2005-03-1000 16219802 PMC1895896

[B10] Lobov IB Renard RA Papadopoulos N Gale NW Thurston G Yancopoulos GD Wiegand SJ Delta-like ligand 4 (Dll4) is induced by VEGF as a negative regulator of angiogenic sprouting Proc Natl Acad Sci USA 2007 104 9 3219 24 10.1073/pnas.0611206104 17296940 PMC1805530

[B11] Suchting S Freitas C le Noble F Benedito R Breant C Duarte A Eichmann A The Notch ligand Delta-like 4 negatively regulates endothelial tip cell formation and vessel branching Proc Natl Acad Sci USA 2007 104 9 3225 30 10.1073/pnas.0611177104 17296941 PMC1805603

[B12] Ridgway J Zhang G Wu Y Stawicki S Liang WC Chanthery Y Kowalski J Watts RJ Callahan C Kasman I Singh M Chien M Tan C Hongo JA de Sauvage F Plowman G Yan M Inhibition of Dll4 signalling inhibits tumour growth by deregulating angiogenesis Nature 2006 444 7122 1083 7 10.1038/nature05313 17183323

[B13] Gerety SS Anderson DJ Cardiovascular ephrinB2 function is essential for embryonic angiogenesis Development 2002 129 6 1397 1410 11880349 10.1242/dev.129.6.1397

[B14] Adams RH Wilkinson GA Weiss C Diella F Gale NW Deutsch U Risau W Klein R Roles of ephrinB ligands and EphB receptors in cardiovascular development: demarcation of arterial/venous domains, vascular morphogenesis, and sprouting angiogenesis Genes Dev 1999 13 3 295 306 10.1101/gad.13.3.295 9990854 PMC316426

[B15] Gerety SS Wang HU Chen ZF Anderson DJ Symmetrical mutant phenotypes of the receptor EphB4 and its specific transmembrane ligand ephrin-B2 in cardiovascular development Mol Cell 1999 4 3 403 414 10.1016/S1097-2765(00)80342-1 10518221

[B16] Kertesz N Krasnoperov V Reddy R Leshanski L Kumar SR Zozulya S Gill PS The soluble extracellular domain of EphB4 (sEphB4) antagonizes EphB4-EphrinB2 interaction, modulates angiogenesis, and inhibits tumor growth Blood 2006 107 6 2330 2338 10.1182/blood-2005-04-1655 16322467 PMC1895726

[B17] Scehnet JS Ley EJ Krasnoperov V Liu R Manchanda PK Sjoberg E Kostecke AP Gupta S Kumar SR Gill PS The role of Ephs, Ephrins, and growth factors in Kaposi sarcoma and implications of EphrinB2 blockade Blood 2009 113 1 254 263 10.1182/blood-2008-02-140020 18836096 PMC2614637

[B18] Hanahan D Heritable formation of pancreatic beta-cell tumours in transgenic mice expressing recombinant insulin/simian virus 40 oncogenes Nature 1985 315 6015 115 122 10.1038/315115a0 2986015

[B19] Bergers G Javaherian K Lo KM Folkman J Hanahan D Effects of angiogenesis inhibitors on multistage carcinogenesis in mice Science 1999 284 5415 808 812 10.1126/science.284.5415.808 10221914

[B20] Yan M Callahan CA Beyer JC Allamneni KP Zhang G Ridgway JB Niessen K Plowman GD Chronic DLL4 blockade induces vascular neoplasms Nature 2010 463 7282 E6 7 10.1038/nature08751 20147986

[B21] Liu R Li X Tulpule A Zhou Y Scehnet JS Zhang S Lee JS Chaudhary PM Jung J Gill PS KSHV-induced notch components render endothelial and mural cell characteristics and cell survival Blood 2010 115 4 887 895 10.1182/blood-2009-08-236745 19965636 PMC2815507

[B22] Yamanda S Ebihara S Asada M Okazaki T Niu K Ebihara T Koyanagi A Yamaguchi N Yagita H Arai H Role of ephrinB2 in nonproductive angiogenesis induced by Delta-like 4 blockade Blood 2009 113 15 3631 3639 10.1182/blood-2008-07-170381 19218547

[B23] Iso T Maeno T Oike Y Yamazaki M Doi H Arai M Kurabayashi M Dll4-selective Notch signaling induces ephrinB2 gene expression in endothelial cells Biochem Biophys Res Commun 2006 341 3 708 14 10.1016/j.bbrc.2006.01.020 16430858

[B24] Hainaud P Contreres JO Villemain A Liu LX Plouet J Tobelem G Dupuy E The role of the vascular endothelial growth factor-Delta-like 4 ligand/Notch4-ephrin B2 cascade in tumor vessel remodeling and endothelial cell functions Cancer Res 2006 66 17 8501 10 10.1158/0008-5472.CAN-05-4226 16951162

[B25] Sawamiphak S Seidel S Essmann CL Wilkinson GA Pitulescu ME Acker T Acker-Palmer A Ephrin-B2 regulates VEGFR2 function in developmental and tumour angiogenesis Nature 2010 465 7297 487 491 10.1038/nature08995 20445540

[B26] Wang Y Nakayama M Pitulescu ME Schmidt TS Bochenek ML Sakakibara A Adams S Davy A Deutsch U Luthi U Barberis A Benjamin LE Makinen T Nobes CD Adams RH Ephrin-B2 controls VEGF-induced angiogenesis and lymphangiogenesis Nature 2010 465 7297 483 486 10.1038/nature09002 20445537

[B27] Iwatsubo T The gamma-secretase complex: machinery for intramembrane proteolysis Curr Opin Neurobiol 2004 14 3 379 383 10.1016/j.conb.2004.05.010 15194119

[B28] Selkoe D Kopan R Notch and Presenilin: regulated intramembrane proteolysis links development and degeneration Annu Rev Neurosci 2003 26 565 597 10.1146/annurev.neuro.26.041002.131334 12730322

[B29] Domenga V Fardoux P Lacombe P Monet M Maciazek J Krebs LT Klonjkowski B Berrou E Mericskay M Li Z Tournier-Lasserve E Gridley T Joutel A Notch3 is required for arterial identity and maturation of vascular smooth muscle cells Genes Dev 2004 18 22 2730 2735 10.1101/gad.308904 15545631 PMC528893

[B30] Liu H Kennard S Lilly B NOTCH3 expression is induced in mural cells through an autoregulatory loop that requires endothelial-expressed JAGGED1 Circ Res 2009 104 4 466 475 10.1161/CIRCRESAHA.108.184846 19150886 PMC2747310

[B31] Armulik A Abramsson A Betsholtz C Endothelial/pericyte interactions Circ Res 2005 97 6 512 523 10.1161/01.RES.0000182903.16652.d7 16166562

[B32] Bergers G Song S Meyer-Morse N Bergsland E Hanahan D Benefits of targeting both pericytes and endothelial cells in the tumor vasculature with kinase inhibitors J Clin Invest 2003 111 9 1287 1295 12727920 10.1172/JCI17929 PMC154450

[B33] Abramsson A Lindblom P Betsholtz C Endothelial and nonendothelial sources of PDGF-B regulate pericyte recruitment and influence vascular pattern formation in tumors J Clin Invest 2003 112 8 1142 1151 14561699 10.1172/JCI18549 PMC213487

[B34] Shaheen RM Tseng WW Davis DW Liu W Reinmuth N Vellagas R Wieczorek AA Ogura Y McConkey DJ Drazan KE Bucana CD McMahon G Ellis LM Tyrosine kinase inhibition of multiple angiogenic growth factor receptors improves survival in mice bearing colon cancer liver metastases by inhibition of endothelial cell survival mechanisms Cancer Res 2001 61 4 1464 1468 11245452

[B35] Peters KG Kontos CD Lin PC Wong AL Rao P Huang L Dewhirst MW Sankar S Functional significance of Tie2 signaling in the adult vasculature Recent Prog Horm Res 2004 59 51 71 10.1210/rp.59.1.51 14749497

[B36] Sato TN Tozawa Y Deutsch U Wolburg-Buchholz K Fujiwara Y Gendron-Maguire M Gridley T Wolburg H Risau W Qin Y Distinct roles of the receptor tyrosine kinases Tie-1 and Tie-2 in blood vessel formation Nature 1995 376 6535 70 74 10.1038/376070a0 7596437

[B37] Foo SS Turner CJ Adams S Compagni A Aubyn D Kogata N Lindblom P Shani M Zicha D Adams RH Ephrin-B2 controls cell motility and adhesion during blood-vessel-wall assembly Cell 2006 124 1 161 173 10.1016/j.cell.2005.10.034 16413489

[B38] Hamzah J Jugold M Kiessling F Rigby P Manzur M Marti HH Rabie T Kaden S Grone HJ Hammerling GJ Arnold B Ganss R Vascular normalization in Rgs5-deficient tumours promotes immune destruction Nature 2008 453 7193 410 414 10.1038/nature06868 18418378

[B39] Jin Y An X Ye Z Cully B Wu J Li J RGS5, a hypoxia-inducible apoptotic stimulator in endothelial cells J Biol Chem 2009 284 35 23436 23443 10.1074/jbc.M109.032664 19564336 PMC2749117

