# Supplementary material for: Combination of Dll4/Notch and Ephrin-B2/EphB4 targeted therapy is highly effective in disrupting tumor angiogenesis
Source: BMC Cancer. 2010 Nov 23;10:641. doi: 10.1186/1471-2407-10-641 (PMC3001720; doi:10.1186/1471-2407-10-641)
Supplement: Additional file 1 — Upregulation of Rgs5 and PSENEN by sEphB4-Alb. Upregulation of Rgs5 and PSENEN in sEphB4-Alb treated RT2 mice was confirmed by quantitative RT-PCR. Regulation of Rgs5 by sEphB4-Alb was also investigated in smooth muscle cells co-cultured with endothelial cells. [file 1471-2407-10-641-S1.PPT]

## Slide 1
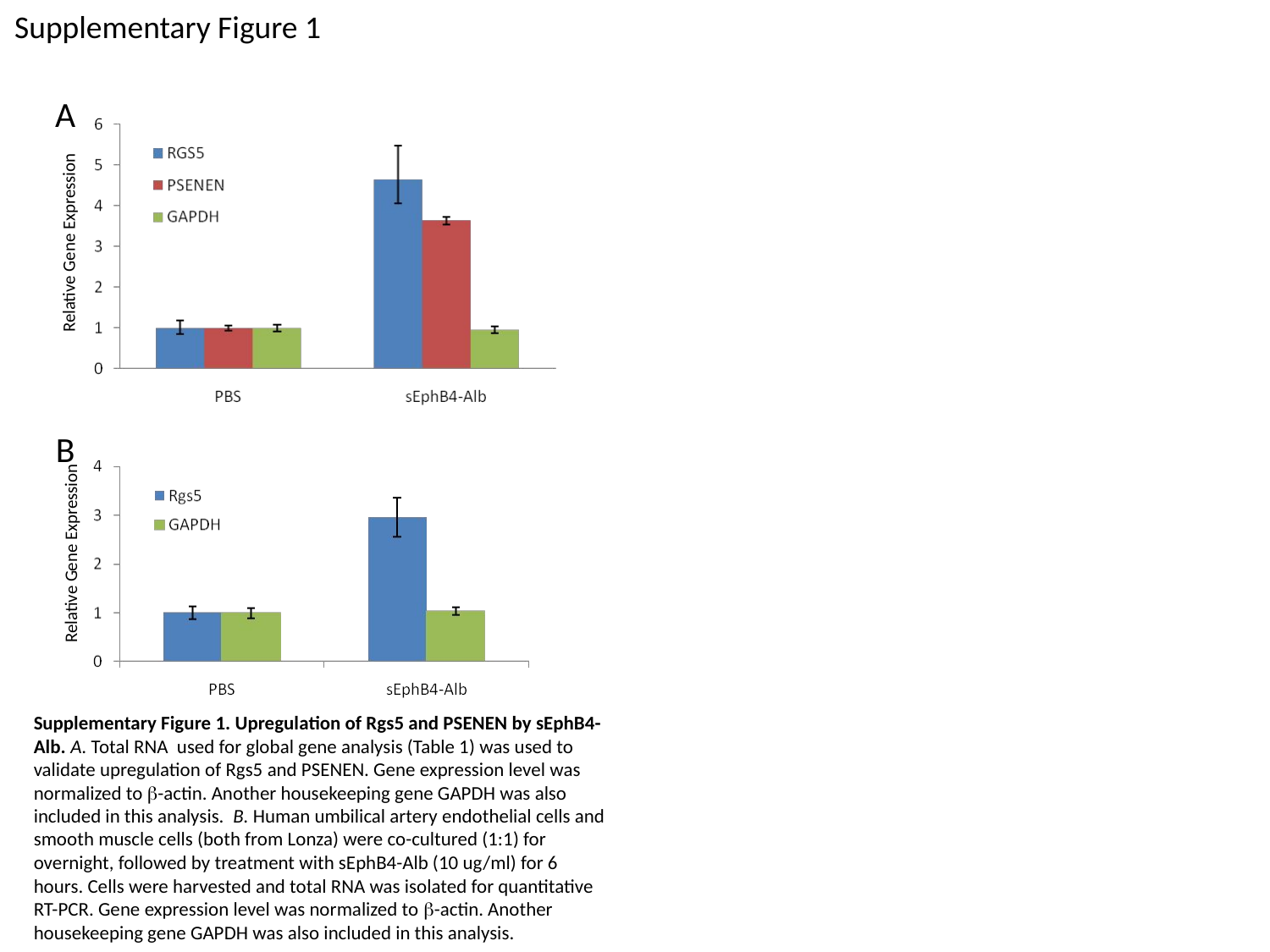

Supplementary Figure 1
A
Relative Gene Expression
B
Relative Gene Expression
Supplementary Figure 1. Upregulation of Rgs5 and PSENEN by sEphB4-Alb. A. Total RNA used for global gene analysis (Table 1) was used to validate upregulation of Rgs5 and PSENEN. Gene expression level was normalized to β-actin. Another housekeeping gene GAPDH was also included in this analysis. B. Human umbilical artery endothelial cells and smooth muscle cells (both from Lonza) were co-cultured (1:1) for overnight, followed by treatment with sEphB4-Alb (10 ug/ml) for 6 hours. Cells were harvested and total RNA was isolated for quantitative RT-PCR. Gene expression level was normalized to β-actin. Another housekeeping gene GAPDH was also included in this analysis.
